# Tumor inhibitory T cell immunity may be largely a transplantation artifact not necessarily dependent upon a lack of Tregs

**DOI:** 10.1186/1742-4682-10-42

**Published:** 2013-06-25

**Authors:** Richmond T Prehn, Liisa M Prehn

**Affiliations:** 1 Department of Pathology, University of Washington School of Medicine, Seattle 98118, USA

## Abstract

There exists a very large literature suggesting that T cells come in a variety of species and that without the action of Tregs tumors would seldom survive inhibition by T cell effectors. We believe that much of the evidence supporting the role of Tregs in cancer is compatible with a perhaps simpler hypothesis based upon the demonstration that that small quantities of effector T cells tend to stimulate tumors while larger quantities of seemingly the same cells are inhibitory (an hormesis-like effect). This possibility seems to destroy much of the need to postulate a role for T cell suppressors (Tregs) in cancer, but the exposure of effector T cells to antigen may convert them into Tregs (Tregs do exist). Furthermore, many other data suggest the possibility that immune inhibition of cancer could be a laboratory artifact seldom if ever seen in unmodified nature.

## The Treg hypothesis

In the cases of both tumor and normal tissues, Foxp3(+)CD25(+)CD4 regulatory T cells (Tregs) have been thought to be of the essence and are the subject of an extensive literature. Tregs with other antigenic specificities have been described. The basic experiment upon which much of the entire edifice of the Treg cell in cancer appears to have been built is described by North as follows: mice grow tumors because the tumor bearer develops Treg cells that interfere with the T effector cell immune inhibition that might, in the absence of the Tregs, have largely prevented tumor growth [1]. The general Treg thesis is supported by experiments demonstrating that effector T cells often do not inhibit tumor growth in immunodepressed mice when the latter are restored with T cells from both immune and tumor-bearing donors. In contrast, immunodepressed control animals, restored only with normal immune cells, often do not grow that same original tumor [1]. Furthermore, if a tumor is highly immunogenic, it can often be made to regress if the animal is heavily irradiated [2]. This result is supposedly attributable to the unique sensitivity of the Treg cells to ionizing radiation, leaving the T effector population relatively intact. The elimination of Tregs by CTLA-4-blocking antibodies has also demonstrated efficacy in various murine models [3,4].

## The immunostimulation phenomenon

It seems there is a logical alternative explanation for most of the observations that seem to necessitate the existence of Tregs, an explanation that need not involve Treg cells at all. The argument we shall now advance does not rule out Tregs, but does, we think, offer an alternative explanation for many, perhaps all, of the observations that have, heretofore, been blamed on those pesky Tregs. We shall begin by quoting from the abstract of a paper that appeared in 1972:

*“Various numbers of spleen cells from specifically immunized mice were mixed with constant numbers of target antigenic tumor cells, and were then inoculated subcutaneously into thymectomized, X-irradiated recipients. Small numbers of admixed immune spleen cells produced a statistically significant, and reproducible, acceleration of tumor growth in the inoculum as compared with controls containing either non immune spleen cells or spleen cells from animals immune to a different, non cross-reacting tumor. Larger numbers of specifically immune spleen cells, however, produced inhibition of the admixed tumor’s growth. These data imply that the normal immune reaction may have a dual function in relation to neoplasia: (i) stimulation of tumor growth, early in the course of the disease, or whenever the immune reaction is minimal; (ii) inhibition of tumor growth in other circumstances*[5]*(Prehn 1972)”.*

It should be noted that in this experiment the stimulatory and inhibitory spleen cells were derived from one and the same population so were qualitatively identical, in contrast to the Tregs usually described in various other experiments. The possibility does persist that the immune cells in the stimulatory and inhibitory populations in the 1972 experiment might not have remained identical after exposure to differing quantities of antigen [5].

**Figure 1:**
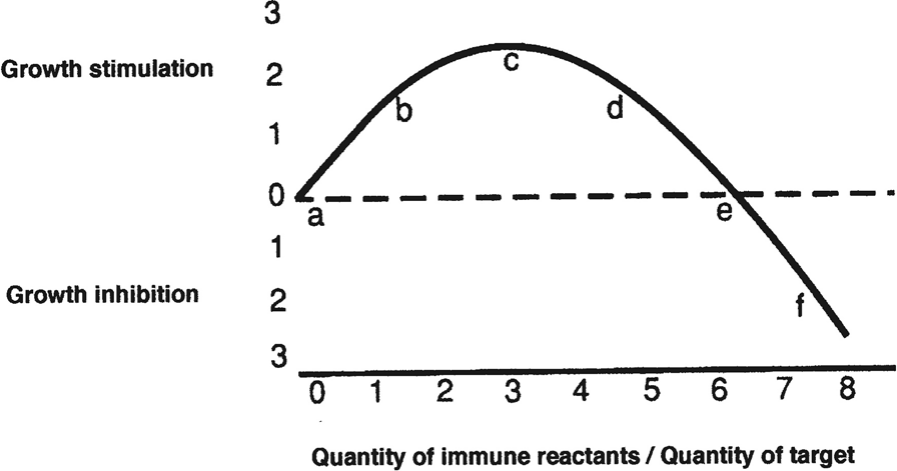
**Idealized chart of data from [**[5]**].**

Figure 1, which has been published previously, depicts an idealized version of the results obtained in [5]. The letters and numerals are only arbitrary aids to facilitate discussion.

Numerous authors have supported these observations [6-11] and it seems there now can be no rational discussion of tumor immunity without asking, “where on the immunostimulation curve do the data lie?”. It is not known whether these same observations apply to immune tolerance to normal tissues and the problems of autoimmunity, but it seems safe to infer that they will have some applicability [12].

Despite the numerous other influences on tumor immune effects that have been noted in the literature [13], the immune reaction curve (Figure 1) seems to us to be of central importance [14] and may apply to all facets of immunity including antibody mediated as well as T cell mediated phenomena [12].

### Blockage of immune versus blockage of non immune inhibition of tumor growth

It has not been determined whether the quantity of immunity as depicted in the figure should designate cell numbers or, more likely, whether the quantity of immunity should be measured by the number of and quality of reactive immune sites. When one examines the apparent action of Tregs in permitting tumor growth, one must ask whether or not the effect seen is caused by dilution (reduction) of normal effector T cells to stimulatory levels rather than by the inhibition of the function of the normal effector T cells by the putative Tregs. It seems that in any situation in which an alteration of tumor growth is attributed to Tregs, it might as easily be attributed to an alteration in T effector cell concentration. As an example, the cure of an immunogenic tumor by radiation of the host could be explained by depriving the tumor of necessary lymphoid stimulation by shifting the total effective immune reaction far enough to the left. (According to this hypothetical scheme, tumors will grow relatively poorly if the immune reaction is shifted sufficiently far to either the left or to the right - see Figure 1). There is experimental evidence by one of our students showing that 3-methylcholanthrene-induced mouse skin papillomas can regress without malignant transformation even when the host animal had been severely immunodepressed; transformation to malignancy as well as growth may depend upon a stimulatory level of immune reaction [15,16].

The possibility that effector T cells are turned into effective Tregs by their exposure to low levels of antigen is an explanation that must not be forgotten. However, somewhat against this idea is the observation that Tregs can sometimes be specific for elements of the normal tissue in which the tumor had originated rather than for the tumor *per se*[17,18]. The possibility exists that Tregs may have been designed by nature to react with normal (non-tumorous) antigens and might thus be involved in the prevention of autoimmunity.

It can now be argued that all untransplanted tumors may be continuously stimulated by an immune response. Inhibitory immunity may be an artifact usually seen only in transplanted tumors or under conditions in which the immune reaction is in some way artificially altered. This argument is suggested by the following facts:

(1) It is relatively difficult to induce growth-inhibiting immunity in the autochthonous mouse to its own native tumor [19].

(2) Cancer progression in the mouse appears to depend upon the immune response [20].

(3) 3-methylcholanthrene-induced mouse skin papillomas apparently fail to transform to malignancy in the absence of immunity [16];

(4) All cancers appear to possess tumor specific antigens [9],

(5) Human carcinomas tend to “flare” in HIV/AIDS patients during and as a result of HAART treatment [21,22].

Selective pressure might suffice to keep most reactions near “c” on the immunostimulation curve. However, some tumors appear to grow better [23,24] or worse [25] when the autochthonous patient is immunodepressed; perhaps in these cases the tumors were still too near their incipiencies at the time of the immune depression for the reaction to have reached full and stable equilibrium near “c” in Figure 1.

## Conclusion # 1

We conclude that it is probable that all cancers have tumor specific antigens and indeed that they probably could only grow *in vivo* with great difficulty in the absence of at least a low level of immune reaction. It may be unnecessary to always postulate the existence of Tregs inasmuch as immunostimulation by effector T cells seems a simpler hypothesis and seems to be an adequate explanation for many of the observations upon which the existence of Tregs appears to depend. However, that effector T cells might be converted into Tregs by exposure to tumor antigen seems probable [5].

## Conclusion # 2

**Figure 2:**
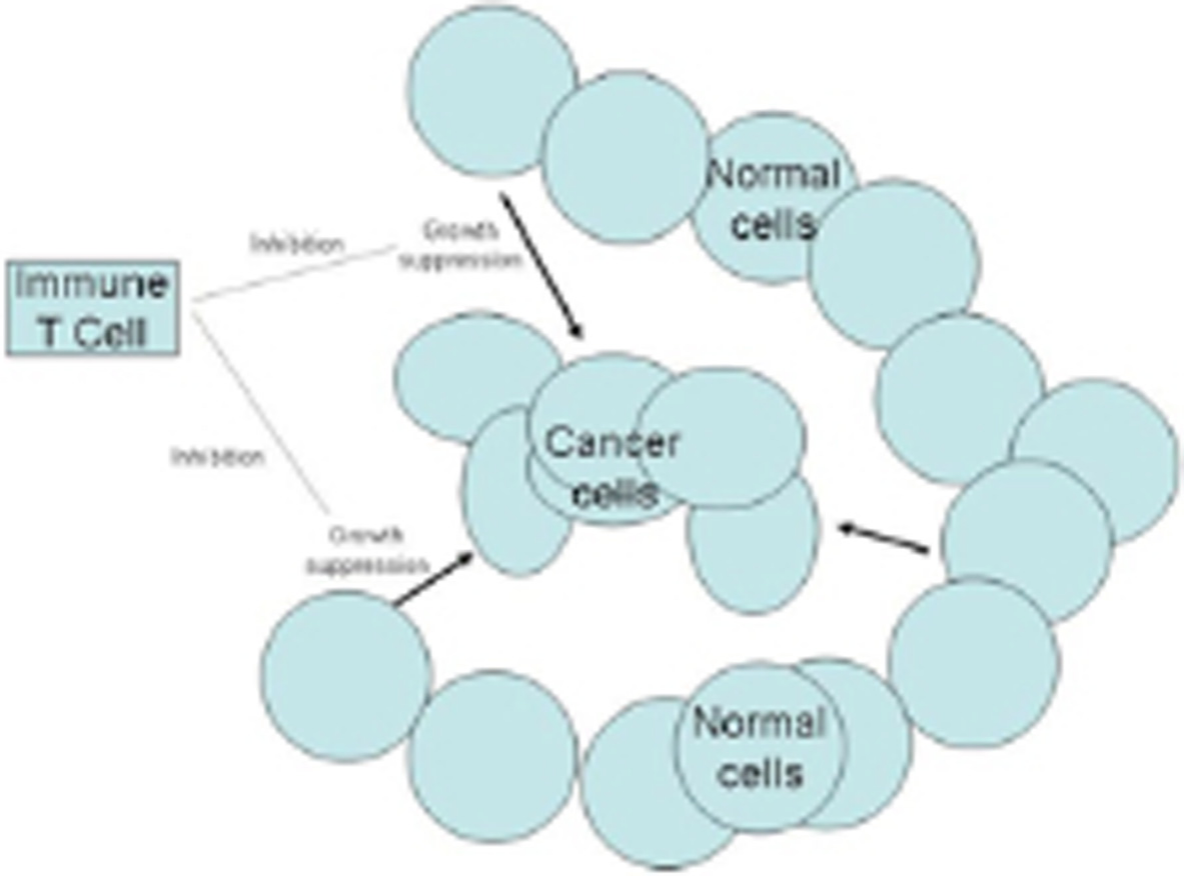
Possible scheme for the mechanism of immune-stimulation of tumor growth.

The facts suggest that a tumor inhibiting immunity is probably a laboratory artifact seldom, if ever, to be found in unaided nature. However, if one believes in the Sonnenschein thesis (which we do), growth and mutiplication are the default conditions of all living cells [26]. Therefore any cell, including a cancerous cell, that fails to grow is being inhibited by some environmental influence. Untransplanted cancers are usually and perhaps continuously stimulated rather than inhibited by the “immune” reaction engendered by them The mechanism of the stimulation could well be an interference by immune T cells of the tumor inhibition provided by the surrounding normal tissue environment [27] (see Figure 2).

A possible variation on this theme could be that so-called Tregs might function to prevent the inhibition of tumor growth by interfering with the antitumor action of the tumor surround [27,28].

## Competing interests

The authors declare that they have no competing interests. Both authors participated equally.
